# Factors associated with mortality from tuberculosis in Iran: an application of a generalized estimating equation-based zero-inflated negative binomial model to national registry data

**DOI:** 10.4178/epih.e2019032

**Published:** 2019-07-09

**Authors:** Fatemeh Sarvi, Abbas Moghimbeigi, Hossein Mahjub, Mahshid Nasehi, Mahmoud Khodadost

**Affiliations:** 1 Department of Biostatistics, School of Public Health, Hamadan University of Medical Sciences, Hamadan, Iran; 2 Modeling of Noncommunicable Diseases Research Center, Department of Biostatistics, School of Public Health, Hamadan University of Medical Sciences, Hamadan, Iran; 3 Research Center for Health Sciences, Department of Biostatistics, Faculty of Public Health, Hamadan University of Medical Sciences, Hamadan, Iran; 4 Center for Communicable Diseases Control, Ministry of Health and Medical Education, Tehran, Iran; 5 Department of Epidemiology and Biostatistics, School of Public Health, Iran University of Medical Sciences, Tehran, Iran; 6 Department of Epidemiology, School of Public Health, Shahid Beheshti University of Medical Sciences, Tehran, Iran

**Keywords:** Tuberculosis, Risk factors, Socioeconomic, Zero-inflated models, Generalized estimating equations

## Abstract

**OBJECTIVES:**

Tuberculosis (TB) is a global public health problem that causes morbidity and mortality in millions of people per year. The purpose of this study was to examine the relationship of potential risk factors with TB mortality in Iran.

**METHODS:**

This cross-sectional study was performed on 9,151 patients with TB from March 2017 to March 2018 in Iran. Data were gathered from all 429 counties of Iran by the Ministry of Health and Medical Education and Statistical Center of Iran. In this study, a generalized estimating equation-based zero-inflated negative binomial model was used to determine the effect of related factors on TB mortality at the community level. For data analysis, R version 3.4.2 was used with the relevant packages.

**RESULTS:**

The risk of mortality from TB was found to increase with the unemployment rate ($\hat{\beta }$=0.02), illiteracy ($\hat{\beta }$=0.04), household density per residential unit ($\hat{\beta }$=1.29), distance between the center of the county and the provincial capital ($\hat{\beta }$=0.03), and urbanization ($\hat{\beta }$=0.81). The following other risk factors for TB mortality were identified: diabetes ($\hat{\beta }$=0.02), human immunodeficiency virus infection ($\hat{\beta }$=0.04), infection with TB in the most recent 2 years ($\hat{\beta }$=0.07), injection drug use ($\hat{\beta }$=0.07), long-term corticosteroid use ($\hat{\beta }$=0.09), malignant diseases ($\hat{\beta }$=0.09), chronic kidney disease ($\hat{\beta }$=0.32), gastrectomy ($\hat{\beta }$=0.50), chronic malnutrition ($\hat{\beta }$=0.38), and a body mass index more than 10% under the ideal weight ($\hat{\beta }$=0.01). However, silicosis had no effect.

**CONCLUSIONS:**

The results of this study provide useful information on risk factors for mortality from TB.

## INTRODUCTION

Tuberculosis (TB) is a global public health problem that causes morbidity and mortality in millions of people each year [1]. In 2017, 10 million people contracted TB, and 1.6 million died from the disease (including 0.3 million people with human immunodeficiency virus [HIV]) [2]. This infectious disease typically involves the lungs (pulmonary TB), but can also involve other sites of the body (extrapulmonary TB).

One of the most important risk factors for TB is poverty. In this context, it has been shown that in households who were poor relative to the general population, the risk of TB infection was about 22% higher. The inability to pay for TB treatment–related costs increased this risk to 36% in households living below the poverty line [3]. Accordingly, low-income and middle-income countries have been found to account for about 95% of TB cases [4], with 6% of the cases in the eastern Mediterranean region occurring in Iran [5]. The effects of poverty and social development indicators (e.g., socioeconomic indicators) on TB have been explored in various studies. Some studies have reported a higher TB prevalence in urban populations [6], and in populations with high levels of immigration [7], populations with low and moderate income [8], areas of high household density per residential unit, and other groups defined by various socioeconomic factors [6]. Other potential risk factors such as diabetes, HIV infection, malnourishment, and silicosis can increase the risk of developing TB. Various studies have explored this issue and reported valuable results, including confirmation of the effects of HIV infection [9,10], low body mass index (BMI) [11], diabetes [12], smoking [13], poverty and malnourishment [14], and alcohol use on TB. Several studies have also reported that the risk of TB mortality was closely related to the presence of diseases such as HIV, silicosis, and diabetes, as well as individual and social characteristics [15,16].

Many of the abovementioned studies examined the relationships between the mentioned risk factors and the risk of TB, while limited articles have examined the effects of these factors on mortality from TB.

It is important for a comprehensive study to be conducted at the national level to determine the relationships of socioeconomic indicators, as well as other potential risk factors, with mortality from TB in Iran. To address this issue, the aim of this study was to investigate the relationships of socioeconomic indicators and other potential risk factors with mortality caused by TB (pulmonary and extrapulmonary) in Iran using a generalized estimating equation (GEE)-based zero-inflated negative binomial (ZINB) model with national registry data.

## MATERIALS AND METHODS

This cross-sectional study was conducted in Iran from March 2017 to March 2018 based on data from the National Tuberculosis and Leprosy Registration Center of Iran’s Ministry of Health and Medical Education (MOHME).

### Participants

The participants were active TB cases (pulmonary and extrapulmonary) who had been diagnosed and registered at the National Tuberculosis and Leprosy Registration Center of the MOHME by 61 universities of medical sciences covering the 429 counties of the 31 provinces of Iran from March 2017 to March 2018. The focus in this study was on TB mortality—defined as deaths among notified TB cases—at the county level during the survey period.

### Questionnaire and software design

The Tuberculosis and Leprosy Control Office is the section of the MOHME that is responsible for registering, analyzing, and controlling TB morbidity, mortality, and its related risk factors in areas of national coverage. A unique computerized questionnaire was used to ensure uniform portal data collection at the level of the 429 counties in the 31 provinces of Iran. The questionnaire included demographic information such as age, gender, BMI, Iranian or non-Iranian nationality, and rural or urban residence of the patients. Information about possible TB risk factors was also gathered, including a history of HIV infection, whether the TB infection occurred in the most recent 2 years, a history of injection drug use (IDU), diabetes, silicosis, long treatment with corticosteroids, malignant disease, chronic kidney failure, gastrectomy and intestinal bypass, chronic malabsorption syndrome, and the patient’s underweight status (more than 10% under the ideal weight).

In the second step, information about the socioeconomic indicators of every county of Iran was obtained from the Statistics Center of Iran. This information included the unemployment rate (the ratio of the unemployed to the active population of at least 10 years of age, multiplied by 100), the immigration rate (the ratio of the difference between immigrants who arrived in the region and the emigrants who left in the target population, multiplied by 100), the illiteracy rate (the proportion of people over 6 years of age with the ability to read and write divided by the total population over 6 years of age in the area, multiplied by 100), household density per residential unit (the number of households in a district divided by the total number of residential units located in the same area), the density of physicians (the number of physicians in a region divided by the population of that area), the urbanization index (the number of population living in urban areas divided by the total population of the region), population density (the ratio of the population in a region to its area), and the distance between the center of the county and the provincial capital (km).

### Statistical analysis

**Figure 1.**
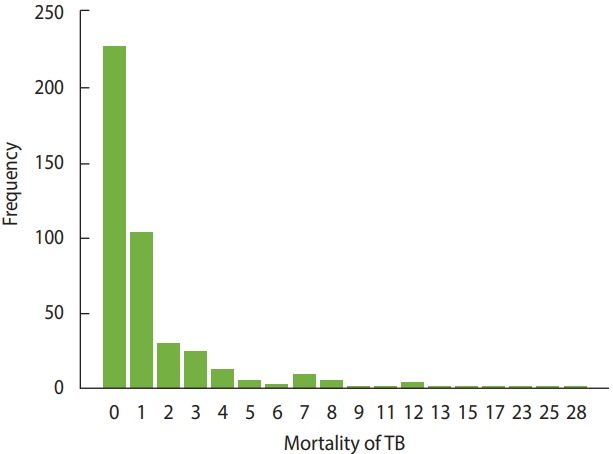
The number of deaths from tuberculosis (TB) in Iran.

In this study, the number of deaths from TB at the county level was considered as the dependent variable, and provinces were considered as clusters that included subjects (i.e., counties). Therefore, the existence of a correlation between counties’ responses within a province is not unexpected. To address this correlation, it was necessary to use an appropriate statistical model, such as a GEE model. However, as shown in Figure 1, in the majority of counties (52.9%), the number of deaths from TB was 0. Furthermore, the score test for zero inflation in multilevel count data [17] and the score test for extra zeros in negative binomial mixed models [18] confirmed the existence of extra zeros in the modeling count data in this study (p<0.001). The usual models that are applied for count data, such as the Poisson or negative binomial models, do not yield credible estimates in the presence of extra zeros. Therefore, the choice was made to use an appropriate model (such as the ZINB model) that would cover extra or inflated zeros and yield more reliable results.

Therefore, due to the presence of correlations between counties within provinces and extra zeros in the data, we used a GEE-based ZINB model, regarding the number of TB patients of every county as an offset term. This model was proposed by Kong et al. [19] in 2015 for handling clustered count data with extra zeros and is used to describe relationships at the population level [20].

For the data analysis, R version 3.4.2 was used with the relevant packages (https://cran.r-project.org/bin/windows/base/old/3.4.2/). The significance level was set at 0.05.

### Ethics statement

This study was performed after receiving approval from the Ethics Committee of Hamadan University of Medical Sciences and was conducted with confidentiality regarding patients’ name and surname.

## RESULTS

In this study, 9,151 patients with active TB (pulmonary and extrapulmonary) from 429 counties of 31 provinces who were registered at the National Tuberculosis and Leprosy Registration Center of the MOHME of Iran were included. Among these notified patients, 606 deaths occurred through the end of the survey period.

The mean age of the TB patients and the patients who died from TB was 46.21±19.24 years and 66.23±51.20 years, respectively. A higher frequency of active TB infections and TB mortality was found in urban regions and in pulmonary TB patients. Additional characteristics of the TB patients and those who died from TB are shown in Table 1.

The aggregate measures of some socioeconomic variables at the county level of Iran are presented in Table 2.

The prevalence of HIV/AIDS in the TB patients and those who died from TB was 3.1% (n=281) and 3.3% (n=20), respectively. Furthermore, 214 (2.3%) of the TB patients and 16 (2.6%) of those who died from TB had a history of IDU (Table 3). More details about the distribution of potential TB risk factors among the TB patients and those who died from TB are shown in Table 3.

The results of fitting the GEE-based ZINB model on socioeconomic indicators and risk factors for TB are presented in Table 4.

In the GEE-ZINB count component, at a level of 0.05, significant relationships were found for the unemployment rate, illiteracy rate, and household density per residential unit with the mortality rate from TB (Table 4). At the level of 0.1, significant relationships with the risk of TB mortality were found for the urbanization level and the distance between the center of the county and the provincial capital.

Furthermore, the results showed that infection with HIV, infection with TB in the last 2 years, IDU, diabetes, underweight, chronic malabsorption syndrome, gastrectomy and intestinal bypass, chronic kidney failure, and long treatment with corticosteroids had significant effects on increasing the risk of mortality from TB. However, no such effect was found for silicosis. Furthermore, in the zero part of the model, underweight (more than 10% less than the ideal weight) was a significant factor. This means that severe weight deficiency had a negative impact on mortality from TB.

In this model, the dispersion parameter and correlation were estimated to be 0.85 and 0.53, respectively, indicating that using the GEE-based ZINB model was an appropriate choice (Table 4).

## DISCUSSION

In this study, we examined socioeconomic and other potential risk factors for TBs mortality in Iran. Except for the immigration rate, physician density, population density, and silicosis, the other factors had significant associations with mortality from TB.

According to our findings, the unemployment rate was associated with an increased risk of TB mortality, in accordance with other studies that have considered this factor [21,22]. Unemployed TB patients may not able to receive the care and treatment necessary for recovery, which can increase their risk of death.

In our study, the illiteracy rate was positively associated with the TB mortality rate. This result is consistent with the findings of de Faria Gomes et al. [23] that the odds ratio of mortality in illiterate individuals was 1.88, in comparison to their literate counterparts. Illiteracy is correlated with less care and delays in diagnosis and treatment, which make death from TB more likely.

With increasing household density per residential unit, the mortality due to TB was increased. This result is similar to the findings of Arcoverde et al. [24], who considered 74 TB patients and found a correlation between residential density and the risk of mortality. Additionally, in higher-density households, transmission of TB was more likely, and it was difficult to provide early detection and appropriate medical care.

The congested population in cities, which increases the probability of contacts, can lead to higher rates of TB morbidity and mortality. In our study, more urbanized areas had a higher TB mortality rate. This result is in accord with the research of Kwan & Ernst [25], who found the urbanization was a major risk factor for TB mortality and morbidity.

Of the other potential risk factors, diabetes, HIV infection, IDU, long-term corticosteroid use, chronic kidney disease, gastrectomy, and underweight (BMI more than 10% under the ideal weight) had a positive relationship with TB mortality, but silicosis had no effect.

Underweight has been reported to be a risk factor for TB-associated mortality and morbidity, due to the impaired cellular immunity associated with low BMI [26]. Various studies have shown that people with a BMI below 18.5 kg/m^2^ are more likely to die from TB than those with a normal BMI [27,28].

A previous meta-analysis showed that diabetes increased the risk of acute pulmonary TB, which may be due to diabetes-related immunosuppression or vitamin A, C, and D deficiency [12]. Several studies have identified diabetes as a risk factor for TB mortality [29,30]. In this study, a significant relationship was found between mortality due to TB and diabetes, implying that diabetes can increase the risk of mortality from TB by impairing immune function.

HIV is one of the strongest known risk factors for TB mortality. Coinfection of TB and HIV dramatically increases the risk of TB mortality; furthermore, HIV and TB coinfection can lead to an increased severity of both conditions, resulting in death [31]. The results of this study also confirmed that HIV increased the risk of TB mortality.

Chronic kidney disease is associated with functional abnormalities in several types of immune cells, such as B cells, T cells, monocytes, neutrophils, and natural killer cells, increasing the risk of TB morbidity and mortality. People with chronic kidney disease are 10 times to 25 times more likely to have active TB than their counterparts [32]. In this study, chronic kidney disease was found to have a significant relationship with the risk of TB mortality.

In the present study, IDU and malnutrition were positively associated with increased mortality from TB. Evidence has suggested that addiction increases the risk of TB mortality [33]. As in the present study, gastrectomy has been found to have a direct effect on mortality and morbidity in other studies, where it was identified as an important risk factor for mortality from TB [33]. The results of this study, along with those of other studies, showed that long-term use of corticosteroids was positively associated with mortality and morbidity from TB [34]. Steroid treatment and further combination therapy with immunosuppressive agents pose a high risk of morbidity, and therefore mortality, from TB. In several previous studies, people with TB who were more frequently exposed to silica dust had a higher mortality rate [35]. However, in the present study, silicosis had no significant relationship with mortality from TB, which may have been because in the population analyzed in this study, the exposure to silica was low.

As in similar studies [36,37], mortality and morbidity from TB in our study were higher in men. Although in some studies, older people showed higher risks of TB-related morbidity and mortality [36], in our study, no significant relationship was found between age and the mortality rate. This finding can be explained by the fact that the mean age of the people with TB who died in every county was entered into our analysis, and that the mean values for age by county were closely distributed.

By identifying the risk factors associated with mortality from TB, the present study provides useful information for planning, prevention, and treatment of TB, with the goal of reducing mortality.

Among the limitations of this study, it should be noted that this study had an observational design, and that more studies are therefore needed to assess causal effects and the real impact of risk factors. Additionally, similar studies found significant relationships of alcohol drinking and smoking with TB incidence [36], but information on those variables was not recorded in our data set, making it impossible for us to examine these relationships. Furthermore, in this study we used a GEE-based ZINB model to determine the related risk factors. However, GEE-based zero inflated generalized Poisson models are a similar framework that can provide more valid and accurate results [38], and the use of this model in similar studies is proposed.

The results of this study determined and considered the most important risk factors for TB mortality in Iran at the national level. Socioeconomic indicators such as unemployment, illiteracy, and household density per residential unit were found to show significant relationships with TB mortality, as were other risk factors such as infection with HIV, a previous history of TB, IDU, and diabetes. Although our results need further investigation, it remains vitally important to reduce the risk factors for mortality from TB.

## Figures and Tables

**Table 1.** Descriptive statistics of study variables among all TB patients, patients who died, and surviving patients

Variables	Total patients (n= 9,151)	Patients who died (n= 606)	Surviving patients (n= 8,545)
Age at diagnosis (yr)	51.72±19.24	66.23±51.20	50.07±20.02
Residency status			
Rural	2,792 (30.5)	165 (27.2)	2,627 (30.7)
Urban	6,359 (69.5)	441 (72.8)	5,918 (69.3)
Nationality			
Iranian	7,789 (85.1)	584 (96.4)	7,204 (84.3)
Non-Iranian	1,362 (14.9)	22 (3.6)	1,340 (15.7)
BMI at diagnosis (kg/m^2^)	19.81±8.88	18.43±8.60	19.94±8.46
Type of TB			
Pulmonary	6,731 (73.5)	534 (88.1)	6,197 (72.5)
Extrapulmonary	2,420 (26.5)	72 (11.9)	2,348 (27.5)
Type of disease			
New case	8,334 (91.1)	594 (98.0)	7,740 (90.6)
Recurrence	292 (3.2)	10 (1.6)	282 (3.3)
Other	525 (5.7)	2 (0.3)	523 (6.1)
Results of treatment			
Cured	1,611 (17.6)	-	-
Died	606 (6.6)	-	-
Treatment failure	98 (1.1)	-	-
Ongoing treatment	4,675 (51.1)	-	-
Other	2,161 (23.6)	-	-

Values are presented as mean±standard deviation or number (%). TB, tuberculosis; BMI, body mass index.

**Table 2.** Descriptive characteristics of socioeconomic variables in Iran at the county level

Socioeconomic variables	Mean±SD^1^	Min-Max
Unemployment rate	12.60±10.72	0.15-67.80
Immigration rate	0.89±7.22	-17.80-33.70
Illiteracy rate	12.95±6.39	2.81-47.46
Household density per residential unit	1.13±0.15	0.73-3.15
Physician density	3.29±52.22	0.00-1,081.20
Urbanization index	0.67±0.19	0.08-0.99
Population density	126.67±560.55	1.00-9,376.00
Distance between the center of the county and provincial capital	131.15±120.94	0.00-897.00

SD, standard deviation; Min, minimum; Max, maximum.

1 Weighted mean.

**Table 3.** Distribution of potential risk factors in all TB patients, patients who died, and surviving patients in Iran

Risk factors	Total patients (n=9,151)	Patients who died (n=606)	Surviving patients (n=8,545)
HIV Infection	281 (3.1)	20 (3.3)	261 (3.0)
Infection with TB in the 2 most recent years	290 (3.2)	16 (2.6)	274 (3.2)
History of IDU	214 (2.3)	16 (2.6)	198 (2.3)
History of diabetes	866 (9.5)	48 (7.9)	818 (9.6)
History of silicosis	13 (0.1)	4 (0.7)	9 (0.1)
Long-term treatment with corticosteroids	113 (1.2)	8 (1.3)	105 (1.2)
Malignant disease	33 (0.4)	1 (0.2)	32 (0.4)
Chronic kidney failure	169 (1.8)	15 (2.5)	154 (1.8)
Gastrectomy and intestinal bypass	20 (0.2)	4 (0.7)	16 (0.2)
Chronic malabsorption syndrome	16 (0.2)	4 (0.7)	12 (0.1)
Underweight^1^	684 (7.5)	60 (9.9)	624 (7.3)

Values are presented as number (%). TB, tuberculosis; HIV, human immunodeficiency virus; IDU, injection drug use.

1 More than 10% under the ideal weight.

**Table 4.** Results of fitting the GEE-based ZINB model of the factors related to TB mortality in Iran

Variables	GEE.ZINB							
	Count component				Zero component			
	ˆβ	SE	z-value	p-value	ˆβ	SE	z-value	p-value
Age^1^	0.00	0.06	0.07	0.09	-15.02	16.92	-0.89	0.81
Immigration rate	0.00	0.01	0.26	0.79	2.68	2.56	1.05	0.29
Unemployment rate	0.02	0.01	2.66	0.007	-2.52	2.64	-0.85	0.39
Illiteracy rate	0.04	0.01	5.00	<0.001	-0.01	0.03	-0.26	0.79
Household density per residential unit	1.29	0.56	2.29	0.02	1.60	0.97	1.65	0.98
Physician density	-0.02	0.01	-1.35	0.18	-12.42	17.44	-0.71	0.48
Urbanization index	0.81	0.45	1.82	0.07	-3.41	2.85	-1.20	0.23
Population density	0.01	0.01	1.07	0.28	0.00	0.00	1.31	0.19
Distance between the center of the county and provincial capital	0.03	0.02	1.70	0.09	-0.01	0.04	0.33	0.74
Infection with HIV	0.04	0.02	1.78	0.07	-5.67	25.53	-0.22	0.82
Infection with TB in the 2 most recent years	0.07	0.02	3.75	<0.001	-9.07	63.20	-0.14	0.88
History of IDU	0.07	0.03	2.34	0.03	-7.95	36.15	-0.22	0.82
Diabetes	0.02	0.01	2.62	0.008	-7.21	34.36	-0.20	0.83
Silicosis	0.00	0.07	0.08	0.93	-15.49	17.01	-0.90	0.37
Underweight (more than 10% under the ideal weight)	0.01	0.00	3.13	0.002	-0.39	0.18	-2.19	0.03
Chronic malabsorption syndrome	0.38	0.11	3.49	<0.001	-8.21	56.12	-0.14	0.88
Gastrectomy and intestinal bypass	0.50	0.11	4.50	<0.001	-0.97	3.40	-0.28	0.77
Chronic kidney failure	0.32	0.08	3.95	<0.001	-0.80	2.58	-0.31	0.75
Long treatment with corticosteroids	0.09	0.04	2.28	0.02	-7.50	37.80	-0.20	0.84
Dispersion	0.85	0.05	17.04	<0.001				
Correlation	0.53							

GEE, generalized estimating equation; ZINB, zero-inflated negative binomial; TB, tuberculosis; SE, standard error; IDU, injection drug use.

1 Mean of age in every county.
